# Phylogenomics of the pantropical Connaraceae: revised infrafamilial classification and the evolution of heterostyly

**DOI:** 10.1007/s00606-024-01909-y

**Published:** 2024-08-03

**Authors:** Jurriaan M. de Vos, Serafin J. R. Streiff, Julien B. Bachelier, Niroshini Epitawalage, Olivier Maurin, Félix Forest, William J. Baker

**Affiliations:** 1 Department of Environmental Sciences - Botany, University of Basel, Schönbeinstrasse 6, 4056 Basel, Switzerland; 2 UMR DIADE, Université de Montpellier, IRD, CIRAD, 911 Avenue Agropolis, 34090 Montpellier, France; 3 Institüt für Biologie/Dahlem Centre of Plant Sciences, Freie Universität Berlin, Altensteinstrasse 6, 14195 Berlin, Germany; 4 Royal Botanic Gardens, Kew, Richmond, Surrey, TW9 3AE UK; 5 The New York Botanical Garden, 2900 Southern Blvd, Bronx, NY 10458 USA; 6 Department of Biology, Aarhus University, Ny Munkegade 116, 8000 Aarhus, Denmark

**Keywords:** Ancient DNA, Angiosperms353, Distyly, Molecular phylogenetics, Oxalidales, Tristyly

## Abstract

Connaraceae is a pantropical family of about 200 species containing lianas and small trees with remarkably diverse floral polymorphisms, including distyly, tristyly, homostyly, and dioecy. To date, relationships within the family have not been investigated using a targeted molecular phylogenetic treatment, severely limiting systematic understanding and reconstruction of trait evolution. Accordingly, their last infrafamilial classification was based only on morphological data. Here, we used phylogenomic data obtained using the Angiosperms353 nuclear target sequence capture probes, sampling all tribes and almost all genera, entirely from herbarium specimens, to revise infrafamilial classification and investigate the evolution of heterostyly. The backbone of the resulting molecular phylogenetic tree is almost entirely resolved. Connaraceae consists of two clades, one containing only the African genus *Manotes* (4 or 5 species), which we newly recognize at the subfamily level. Vegetative and reproductive synapomorphies are proposed for Manotoideae. Within Connaroideae, Connareae is expanded to include the former Jollydoreae. The backbone of Cnestideae, which contains more than half of the Connaraceae species, remains incompletely resolved. Reconstructions of reproductive system evolution are presented that tentatively support tristyly as the ancestral state for the family, with multiple parallel losses, in agreement with previous hypotheses, plus possible re-gains. However, the great diversity of stylar polymorphisms and their phylogenetic lability preclude a definitive answer. Overall, this study reinforces the usefulness of herbarium phylogenomics, and unlocks the reproductive diversity of Connaraceae as a model system for the evolution of complex biological phenomena.

**Supplementary Information:**

The online version contains supplementary material available at 10.1007/s00606-024-01909-y.

## Introduction

**Fig. 1:**
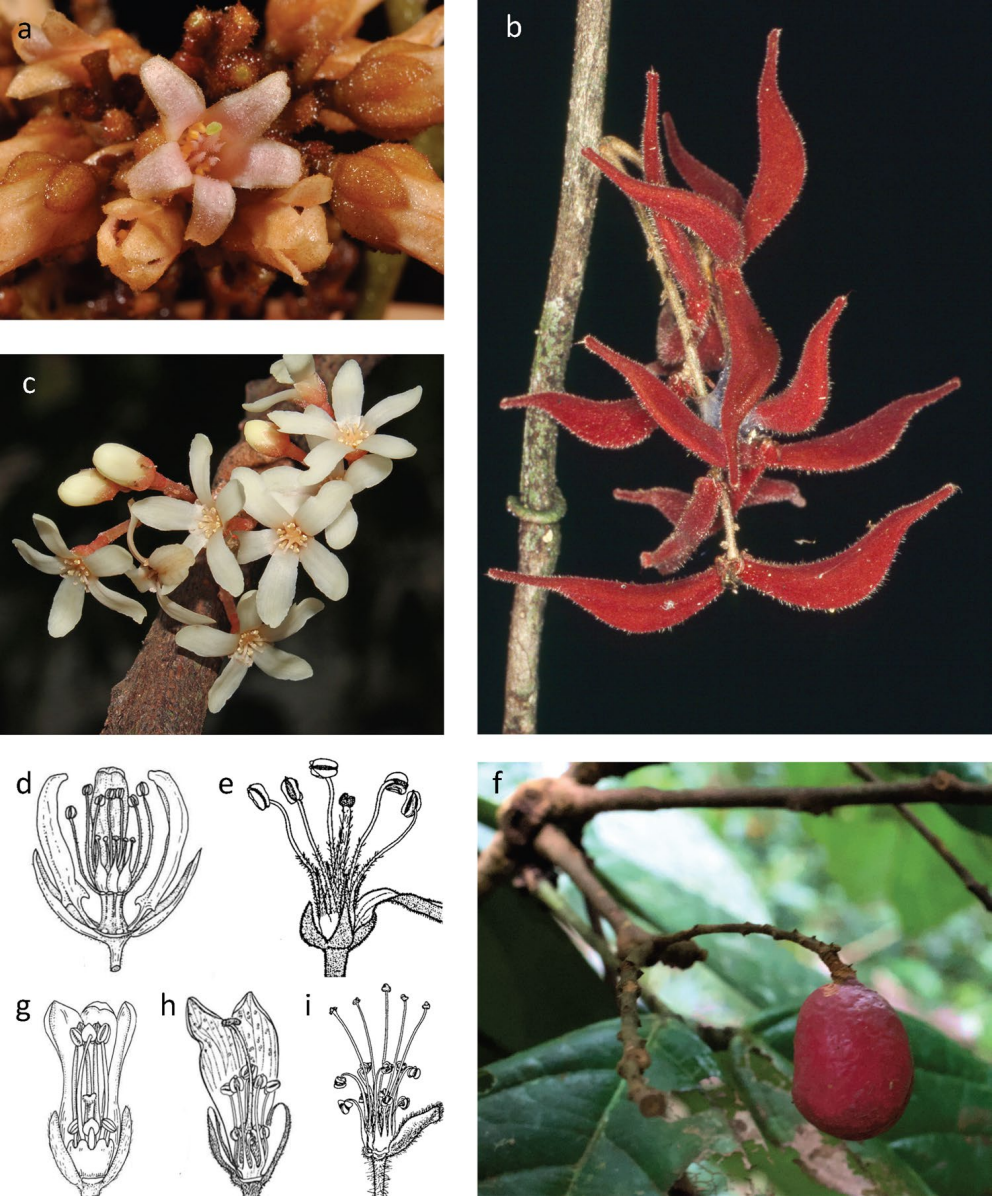
Flowers and fruits of selected Connaraceae species: **a** *Connarus whitfordii* Merr., **b** *Cnestis corniculata* Lam., **c** *Agelaea trinervis* (Llanos) Merr. **d** *Manotes macrantha* (Gilg) G.Schellenb., **e** *Ellipanthus hemandradenioides* Brenan, **f** *Connarus ruber* (Poepp.) Planch., **g** *Jollydora duparquetiana* (Baill.) Pierre, **h** *Connarus africanus* Lam., **i** *Rourea orientalis* Baill. Manotoideae: **d**; Connaroideae-Connareae: **a**, **e**, **f**, **g**, **h**; Connaroideae-Cnestideae: **b**, **c**, **i**. Photo credits: **a** Pelser et al. (2011) onwards; **b** WJB; **c** Kean Mazo; **d**, **e**, **g**–**i**, Breteler (1989); **f** SJRS; reproduced with permission

Connaraceae R.Br. is a pantropical family of large lianas to small trees, with estimates of species numbers and genera ranging from 193 to 385 species in 12–24 genera, largely restricted to tropical forests (Breteler 1989; Forero 1983; Schellenberg 1938). The family is morphologically well-characterized by alternate, exstipulate, imparipinnate to tri- or unifoliolate (or rarely palmate) leaves, and actinomorphic pentamerous flowers with a diplostemonous androecium and a gynoecium with 1 or 5 free carpels, each comprising two ovules and eventually developing into a follicle containing usually a single seed (Lemmens et al. 2004; Fig. 1). A sister relation of Connaraceae to Oxalidaceae within Oxalidales is strongly supported based on early molecular data (Chase et al. 1993) and morphology (Matthews and Endress 2002), a preliminary analysis of phylogenomic data (Baker et al. 2022), and consistent with an Oxalidales-wide analysis (Pillon et al. 2021). In stark contrast to the clear circumscription and position of Connaraceae as a whole, infrafamilial relations remain unclear. Specifically, generic delimitation is rather unstable, as no global revision was performed since Schellenberg (1938). Notwithstanding recent, geographically focused treatments (Toledo et al. 2020, 2024), no molecular phylogenetic analysis is available, and previous authors highlighted conflicting breadth of species concepts between authors working on different continents (e.g., Breteler 1989). Nevertheless, a tribal classification of Connaraceae has been proposed (Lemmens 1989b), including four tribes (Connareae Planch., Jollydoreae (Gilg) Lemmens, Manoteae Lemmens, and Cnestideae Planch.) that were defined based on number of leaflets and their venation, number of carpels, dehiscence, seed number and seed attachment within the follicles, and pollen type. This classification was based on careful cladistic and phenetic analysis of morphology, and strongly differed from the one Schellenberg (1938) proposed (with two subfamilies, one of which with five tribes; Table 1). The latter considered inflorescence architecture as of primary importance and ignored leaf traits and carpel number. Whether the tribes recognized by Lemmens (1989b) represent monophyletic lineages and how they are related remains untested and is critical for a better understanding of trait evolution.

**Table 1:** Identity and reproductive system of Connaraceae and outgroup samples

Current name [sample]*	Generic lineage**	Systematic position (Schellenberg 1938)	Systematic position (Lemmens 1989b)	Continent	Accession***	Specimen	Reprodutive system****	Binary scoring****
*Agelaea paradoxa* Gilg [5326]	*Castanola* Llanos	Connaroideae—Castanoleae	Cnestideae	Africa	ERR7618343	*C.C.H.* *Jongkind* *7486* (WAG.1498392)	Tristyly	Trimorphism
*Agelaea pentagyna* (Lam.) Baill. [5323]	*Agelaea* Sol. ex. Planch. (TYPE)	Connaroideae—Agelaeeae	Cnestideae	Africa, Madagascar	ERR5033663	*Nimba Bot. Team WH 1055* (WAG.1440330)	Tristyly	Trimorphism
*Cnestidium rufescens* Planch. [2875]	*Cnestidium* Planch. (TYPE)	Connaroideae—Connareae	Cnestideae	America	ERR5033322	*Q*. *Jimenez* *1652* (K000648333)	Semihomostyly	Trimorphism
*Cnestis bomiensis* Lemmens [5328]	*Cnestis* Juss.	Connaroideae—Cnestideae	Cnestideae	Africa	ERR7618345	*C.C.H.* *Jongkind* *4475* (WAG.1440479)	NA^2^	NA
*Cnestis ferruginea* Vahl ex DC. [5320]	*Cnestis* Juss.	Connaroideae—Cnestideae	Cnestideae	Africa	ERR5034759	*Haba 80* (WAG.1441230)	Homostyly	NA
*Cnestis palala* (Lour.) Merr. [5846]	*Tysanus* Lour. (TYPE)	Connaroideae—Cnestideae	Cnestideae	Asia	ERR7618540	*K*. *Larsen* *8223* (P05614477)	Semihomostyly	Trimorphism
*Cnestis polyphylla* Lam. [2543]	*Cnestis* Juss. (TYPE)	Connaroideae—Cnestideae	Cnestideae	Africa, Madagascar	ERR4180133	*D.G.A*. *Styles* *2365* (K)	Semihomostyly	Trimorphism
*Cnestis uncata* Lemmens [5845]	*Cnestis* Juss.	Connaroideae—Cnestideae	Cnestideae	Africa	ERR7618539	*F.J*. *Breteler* *8393* (P05613855)	Distyly	Dimorphism
*Pseudoconnarus macrophyllus* Gilg ex G.Schellenb. [3956]	*Pseudoconnarus* Radlk. (TYPE)	Connaroideae—Byrsocarpeae	Cnestideae	America	ERR5034662	*A*. *Vicentini* *1233* (K000648335)	Semihomostyly	Trimorphism
*Rourea acutipetala* Miq. [5843]	*Roureopsis* Planch., *Taeniochlaena* Hook.f.	Connaroideae—Byrsocarpeae	Cnestideae	Asia	ERR7618537	*S*. *Bunkerd* *26* (P06841119)	Distyly	Dimorphism
*Rourea balansana* Baill. [3093]	*Santaloides* L. ex G.Schellenb	Connaroideae—Byrsocarpeae	Cnestideae	Asia	ERR7617971	*Y*. *Pillon* *28* (K)	Distyly	Dimorphism
*Rourea calophylla* (Gilg ex G.Schellenb.) Jongkind [5552]	*Paxia* Gilg	Connaroideae—Byrsocarpeae	Cnestideae	Africa	ERR5034780	*J.* *Schoenmaker* *257* (WAG.1441395)	Distyly	Dimorphism
*Rourea coccinea* (Schumach. & Thonn.) Benth. [5324]	*Byrsocarpus* Schumach. (TYPE), *Jaundea* Gilg	Connaroideae—Byrsocarpeae	Cnestideae	Africa	ERR7618342	*C.C.H*. *Jongkind* *7728* (WAG.1441618)	Distyly	Dimorphism
*Rourea minor* (Gaertn.) Merr. [5329]	*Santaloides* L. ex G.Schellenb. (Africa)	Connaroideae—Byrsocarpeae	Cnestideae	Africa, Asia	ERR9229860	*C.C.H.* *Jongkind* *9266* (WAG.1441914)	Distyly	Dimorphism
*Rourea minor* (Gaertn.) Merr. [2571]	*Santaloides* L. ex G.Schellenb. (Asia)	Connaroideae—Byrsocarpeae	Cnestideae	Africa, Asia	ERR7617900	*M.W*. *Chase* *1221* (K)	Distyly	Dimorphism
*Rourea myriantha* Baill. [5840]	*Paxia* (TYPE)	Connaroideae—Byrsocarpeae	Cnestideae	Africa	ERR7618535	*J.J*. *Bos* *4121* (P05612241)	Distyly	Dimorphism
*Rourea orientalis* Baill. [5321]	*Byrsocarpus* Schumach.	Connaroideae—Byrsocarpeae	Cnestideae	Africa	ERR7618340	*C.C.H.* *Jongkind* *3470* (WAG.1442169)	Distyly	Dimorphism
*Rourea parviflora* Gilg [5318]	*Santaloidella* Schellenberg	Connaroideae—Byrsocarpeae	Cnestideae	Africa	ERR7618338	*J.L.C.H.* *van Valkenburg* *2695* (WAG.1442218)	Tristyly	Trimorphism
*Rourea solanderi* Baker [5322]	*Spiropetalum* Gilg (TYPE)	Connaroideae—Byrsocarpeae	Cnestideae	Africa	ERR7618341	C.C.H. *Jongkind* *2132* (WAG.1442454)	Tristyly	Trimorphism
*Rourea thomsonii* (Baker) Jongkind [5327]	*Jaundea* Gilg	Connaroideae—Byrsocarpeae	Cnestideae	Africa	ERR7618344	*C.C.H. Jongkind* *11261* (WAG.1541466)	Distyly	Dimorphism
*Rourea emarginata* (Jack) Jongkind [3409]	*Roureopsis* Planch.	Connaroideae—Connaroideae	Cnestideae	Asia	ERR7617901	*M.W*. *Chase* *1222* (K)	Distyly	Dimorphism
*Burttia prunoides* Baker f. & Exell [3942]	*Burttia* Bak.f. & Exell (TYPE)	Connaroideae—Connaroideae	Connareae	Africa	ERR7618047	*H.H*. *Schmidt* *1229* (K000648330)	Distyly	Dimorphism
*Connarus africanus* Lam. [5851]	*Connarus* L. (Africa)	Connaroideae—Connareae	Connareae	Africa	ERR5034792	*C.C.H*. *Jongkind* *8130* (P05487889)	Distyly	Dimorphism
*Connarus cochinchinensis* (Baill.) Pierre [5841]	*Connarus* L. (Asia)	Connaroideae—Connareae	Connareae	Asia	ERR7618536	*S*. *Hul* *725* (P06172837)	Distyly^1^	Dimorphism^1^
*Connarus perrottetii* (DC.) Planch. [5844]	*Connarus* L. (S. America)	Connaroideae—Connareae	Connareae	America	ERR7618538	*M.J*. *Jansen-Jacobs* *6128* (P05519995)	Tristyly^1^	Trimorphism^1^
*Connarus thonningii* (DC.) G.Schellenb. [5551]	*Connarus* L. (Africa)	Connaroideae—Connareae	Connareae	Africa	ERR7618362	*C.C.H.* *Jongkind* *2991* (WAG.1382987)	Distyly	Dimorphism
*Ellipanthus beccarii* Pierre [5849]	*Pseudellipanthus* G.Schellenb. (TYPE)	Connaroideae—Castanoleae	Connareae	Asia	ERR7618541	*A*. *Villamil* *314* (P05615654)	Dioecy	NA
*Ellipanthus madagascariensis* (Schellenb.) Capuron ex Keraudren [5847]	*Ellipanthus* Hook.f.	Connaroideae—Castanoleae	Connareae	Madagascar	ERR5034791	*C.Z*. *Rakotonirina* *612* (P00967440)	Distyly	Dimorphism
*Ellipanthus razanatsimae* Randrian. & Lowry [5990]	*Ellipanthus* Hook.f.	Connaroideae—Castanoleae	Connareae	Madagascar	ERR7618573	*A*. *Razanatsima* *378* (MO)	Distyly	Dimorphism
*Hemandradenia* sp. [6608]	*Hemandradenia* Stapf	Connaroideae—Agelaeeae	Connareae	Africa	ERR5084299	*N*. *Hallé* *1727* (P05614608)	Distyly	Dimorphism
*Vismianthus punctatus* Mildbr. [2516]	*Vismianthus* Mildbr. (TYPE)	Connaroideae—Castanoleae	Connareae	Africa	ERR5034653	*S*. *Bidgood* *1365* (K000568982)	Distyly	Dimorphism
*Jollydora armandui* Jongkind [5317]	*Jollydora* Pierre	Jollydoroideae	Jollydoreae	Africa	ERR7618337	*C.C.H. Jongkind* *9914* (WAG0295171)	Tristyly	Trimorphism
*Jollydora duparquetiana* (Baill.) Pierre [5304]	*Jollydora* Pierre, (*Anthagathis* Harms, *Ebandoua* Pellegrin)	Jollydoroideae	Jollydoreae	Africa	ERR7618335	*I*. *Parmentier* *4839* (K)	Tristyly	Trimorphism
*Manotes expansa* Sol. ex Planch. [5325]	*Manotes* Sol. ex Planch. (TYPE)	Connaroideae—Agelaeeae	Manoteae	Africa	ERR5034760	*C.C.H*. *Jongkind* *13423* (WAG.1963700)	Tristyly	Trimorphism
*Manotes macrantha* (Gilg) G.Schellenb. [5319]	*Dinklagea* Gilg (TYPE)	Connaroideae—Agelaeeae	Manoteae	Africa	ERR7618339	C.C.H. *Jongkind* *7088* (WAG.1441318)	Distyly	Dimorphism
*Dapania pentandra* Capuron [5850]	(outgroup)	(Oxalidaceae)	(outgroup)		ERR7618542	*R*. *Razakamalala* *3080* (P05578081)		
*Dapania racemosa* Korth. [2715]	(outgroup)	(Oxalidaceae)	(outgroup)		ERR7617908	*Ambri 1014* (K)		
*Sarcotheca macrophylla* Blume [3964]	(outgroup)	(Oxalidaceae)	(outgroup)		ERR5034663	*P*. *Wilkie* *9542* (K000648329)		

**Fig. 2:**
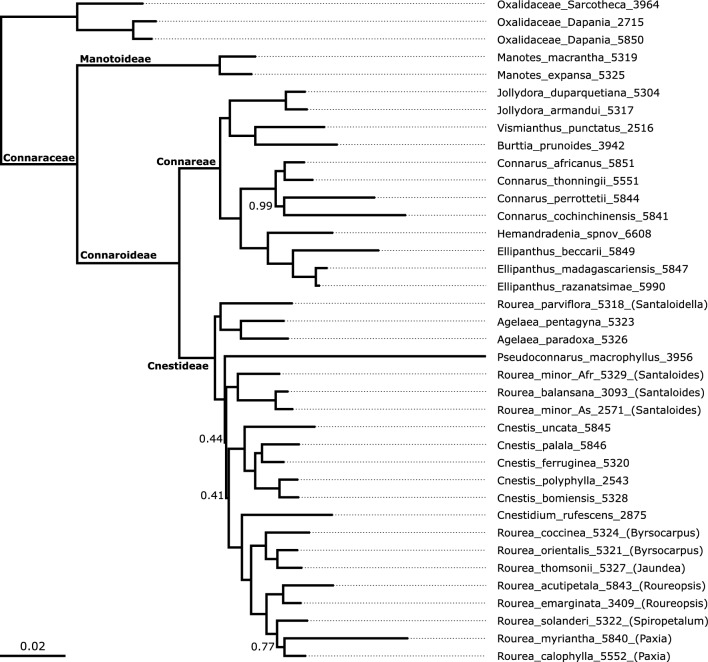
Phylogenetic tree of Connaraceae, with proposed classification indicated, with ASTRAL topology and RAxML branch lengths in substitutions per site (scale bar indicated). All branches had local posterior probability support of 1.0 except where indicated to the left of a branch. Proposed names for subfamilies and tribes are indicated by text above or below their corresponding stem lineage. For species of the polyphyletic *Rourea* sensu Jongkind (1989), former generic names are indicated in brackets (see Table 1)

*Current names follow the generic concepts of Breteler (1989), sample numbers are indicated in Fig. 2. **Generic lineages are names that were at some point recognized prior to Breteler (1989), (TYPE indicates that the sample represents the Type species of the genus name). ***European Nucleotide Archive run accession number, all within Bioproject PRJEB35285. ****Reproductive system scoring: see text; remarks: (1) tentatively scored based on precise floral drawings including a mid-morph flower (Vidal 1962), a long-morph herbarium specimen, plus Lemmens' (1989a) general statement that the short morph is present in American *Connarus*. (2) Reproductive system unknown: the species was described from three specimens that were all short-style morphs

Connaraceae flowers are remarkable for their diversity in floral polymorphisms. In particular, it is one of seven families known to contain a tristylous reproductive system (Naiki 2012; with Amaryllidaceae, Linaceae, Lythraceae, Oxalidaceae, Pontederiaceae and Thymelaeaceae). Tristyly is a form of heterostyly, a genetic polymorphism where plants produce flowers with either two (distyly) or three (tristyly) floral morphs that differ in the reciprocal position of anthers and stamen, typically complemented with a physiological self- and intra-morph incompatibility system and ancillary characters (Barrett 2019). Families in which tristyly occurs often also contain distylous species, but Connaraceae is special in this respect because it contains various types of distyly (lacking either the mid-style or short-style morph of tristyly), homostylous species (that lack a genetic floral polymorphism), dioecy (with morph-specific sex-sterility), and various intermediate forms, including semihomostyly, and polymorphisms of stigmatic surface (Lemmens 1989a).

No other family is as diverse in forms of heterostyly as Connaraceae. This unparalleled diversity is significant because a wide range of phylogenetic studies have attempted to reconstruct transitions between heterostyly and other floral constellations (e.g., Barrett 2019 cites more than 20 examples), for instance because of its relevance to understanding the drivers of the evolution of plant reproductive diversity (Barrett 2019). Phylogenetic evidence on the floral precursor of tristyly, however, remains lacking (for theory, see Charlesworth 1979). Although evolutionary losses of tristyly to less complex and/or selfing forms may be more common than gains (e.g., Kohn et al. 1996, Barrett et al. 2009; Lewis and Rao 1971; Ornduff 1979), tristyly could persist evolutionarily if the rate of its loss is less than the mean speciation rate of tristylous lineages, even if it rarely evolves (Maddison et al. 2007).

Addressing these issues requires developing a robust phylogenetic hypothesis for Connaraceae based on molecular data. Until recently, however, this was a daunting undertaking, because Connaraceae—like many tropical plant families—are logistically challenging to sample in the field due to their pantropical distribution, and the fact that many species do not produce flowers or fruits that are easily inspected from the forest floor. In addition, species identification is in Connaraceae is generally difficult because of the scattered and disjointed taxonomic literature, without a recent global treatment. Recent developments in herbarium phylogenomics, however, have greatly ameliorated this situation (reviewed e.g., by Brewer et al. 2019; Burbano and Gutaker 2023; Dodsworth et al. 2019; Kistler et al. 2020; Baker et al. 2021). First, high-throughput sequencing (as opposed to Sanger sequencing) can successful produce high-quality sequence data from the highly degraded DNA usually obtained from preserved herbarium specimens (Kistler et al. 2020; Raxworthy and Smith 2021). Second, the development of RNA probes to selectively enrich genomic libraries for targeted genes enabled more applications (Dodsworth et al. 2019). Off-the-shelf universal “bait kits” that target genes that work well for phylogenetics across angiosperm lineages circumvent the need to have access to prior genetic information. In particular, the development of the Angiosperms353 probe set (Johnson et al. 2019), allowing selective enrichment of 353 low-copy nuclear genes, is revolutionizing plant phylogenetics, as it is becoming widely adopted (e.g., Brewer et al. 2019; Baker et al. 2021; Maurin et al. 2021; Larridon et al. 2021; Pillon et al. 2021; Hendriks et al. 2023), despite some limitations (Lee et al. 2021). In the case of Connaraceae, these positive developments enable us to exploit existing collections, making it feasible to infer a first molecular phylogenetic tree of this neglected plant family without access to prior sequencing information, circumventing the prohibitively challenging logistics of field sampling.

In this study, we infer a molecular phylogenetic species tree based on Angiosperms353 nuclear gene and chloroplast sequences derived from herbarium specimens spanning the generic diversity of Connaraceae. We focus taxon sampling from expert-determined herbarium specimens of the type species of the many lineages that at various times were treated as accepted genera. We then use the phylogenetic tree to test two hypotheses: the four tribes recognized by Lemmens (1989b) are monophyletic; and tristyly is the ancestral reproductive system in the family, with distyly and other syndromes derived from it. We then present a formal, updated supergeneric classification including a new subfamily. Overall, this integrated systematic study represents considerable progress toward a stable taxonomy of Connaraceae and marks an important step toward unlocking Connaraceae as a model for further systematic and evolutionary studies, including the evolution of reproductive systems.

## Materials and methods

### Taxon sampling

We employed an herbarium-phylogenomic approach with “nomenclatural sampling” to overcome the logistic challenges of sample acquisition for a pantropical clade without a previous phylogenetic framework, while ensuring all major lineages were represented. Specifically, we aimed to include representatives of all genera accepted and all newly synonymized in Breteler (1989), targeting in particular the type species of each generic name from herbarium specimens with an expert determination, or a morphologically similar species. This ensured that our phylogenetic sampling encompasses the morphological breadth of the family, by including all taxa once considered morphologically distinct enough to be qualified at the generic level, while also providing a stable starting point for filling in the phylogenetic tree with more species in later studies. In addition, we included species that represent otherwise unsampled reproductive systems and in some cases included multiple samples for species that by some concepts occur on multiple continents. Overall, after excluding poor quality sequences, we could include 38 samples representing 35 Connaraceae plus three outgroup taxa, including all four tribes accepted by Lemmens (1989b), all 12 genera accepted by Breteler (1989), and a further 8 genera synonymized by him or previous authors. From each specimen, we removed up to ca. 1–2 cm^2^ leaf tissue for molecular analysis.

### Molecular methods

DNA extraction, library preparation, target enrichment, and DNA sequencing follow Baker et al. (2022). Briefly, we extracted DNA using a modified CTAB protocol (Doyle and Doyle 1987), which we fragmented using sonication (Covaris M220 Focused-ultrasonicator with microTUBEs AFA Fiber Pre-Slit Snap-Cap (Woburn, MA, USA) when DNA fragment length exceeded 350 bp. We prepared Dual-indexed libraries for Illumina sequencing using the DNA NEBNext UltraTM II Library Prep Kit at half the recommended volume, with Dual Index Primers Set 1, NEBNext Multiplex Oligos for Illumina (New England BioLabs). After pooling 20–25 DNA libraries (equimolar for a total of 1 μg of DNA), we hybridized them using the Angiosperms353 v1 expert panel (Arbor Biosciences, Ann Arbor, MI, USA; Catalog #308196; Johnson et al. 2019) at 65 °C for 28–32 h. After amplifying enriched products for 10 cycles and cleaning them, we quantified and multiplexed them and then sequenced them on an Illumina MiSeq (v3 reagents, 2 × 300-bp paired-end, Illumina, San Diego, CA, USA) at the Royal Botanic Gardens, Kew, or on an on Illumina HiSeq (2 × 150-bp paired-end reads) at Genewiz (Takeley, UK. Sequencing reads were made publicly available through the European Nucleotide Archive (bioproject PRJEB35285; run accession numbers in Table 1) and included in the Kew Tree of Life explorer (Baker et al. 2022).

### Bioinformatic and phylogenetic methods

Bioinformatic processing and phylogenetic inference was performed on the sciCORE (http://scicore.unibas.ch/) scientific computing center at the University of Basel. We processed raw sequencing reads by removing adapters and trailing low-quality bases using Trimmomatic (Bolger et al. 2014) with default settings. We assembled sequences using the HybPiper v.1.3.1 pipeline (Johnson et al. 2016). For each target gene, it uses BWA (v.0.7.15, Li and Durbin 2009) to select relevant reads using a custom target file (created by selecting Oxalidales sequences from the 'mega353' target file; McLay et al. 2021). We also added targets for three high-copy regions: plastid *rbc*L and *mat*K genes, plus the nuclear ribosomal region downloaded from GenBank; Accession numbers in Electronic Supplementary Table S1, hereafter we refer to all targeted genomic regions as “genes”) and assembles them de-novo using SPAdes (v.3.10.1, Bankevich et al. 2012). We carried the extracted exon sequences forward without attempting to extract intron sequences.

To detect and remove potential paralogous sequences, we filtered reconstructed sequences using HybPhaser (v.2.0, Nauheimer et al. 2021). HybPhaser flags genes that display excessive heterozygosity (which can occur if multiple, somewhat divergent, gene copies are jointly assembled into a single sequence) by back-mapping raw reads to the reconstructed sequences. We selected settings that were stricter than the default and scored a site as heterozygous at a minimum coverage of 6 × and minimum count of 3, using ambiguity coding. We then excluded species for which fewer than 50 genes were reconstructed; it was not necessary to exclude species due to their heterozygosity, as its species-mean value was below 1.1% for all species. This also suggest that none of the species are of recent hybrid origin (Nauheimer et al. 2021). Next, we excluded six genes with > 3% heterozygous sites (i.e., genes “4471”, “5168”, “5434”, “5463”, “6373”, and “6791”) and three that were recovered in less than a third of the species (i.e., genes “6514”, “6148”, and “6557”). In total, we included 346 genes (incl. 343 of the Angiosperms353 set, plus the three high copy genes) and 38 species, including three outgroup taxa from the Oxalidaceae. Overall, the species-by-genes matrix had an occupancy of 96.2% (i.e., we included 12,660 sequences), representing roughly a quarter million bases per species (median 249,732, range 63,558 to 266,643).

For phylogenetic inference, we first aligned each locus using MAFFT (v.7.490, “localpair” option, Katoh and Standley 2013) and computed its maximum likelihood gene tree using RAxML-NG (v.1.1.0, GTR + G substitution model; Kozlov et al. 2019). We then computed a species tree using ASTRAL (v.5.7.7, Zhang et al. 2018) from the gene trees after collapsing branches with near-zero lengths. ASTRAL disassembles gene trees into their constituent quartets and then combines them together such that implied incomplete lineage sorting is minimized, while assuming no reticulate evolution. Local posterior probabilities (pp) at each node (i.e., the fraction of quartets that support the depicted topology among all quartets informative of the node) allow to evaluate support (where pp > 0.95 is “significant”) and support or reject the hypothesis that Lemmens' tribal classification reflects monophyletic units. Terminal branches are of arbitrary length in ASTRAL trees, and therefore, we computed meaningful branch lengths for downstream analyses. Specifically, implemented an approach with custom scripts similar to “gene-shopping” (Smith et al. 2018) and selected among the loci with full taxon sampling the 12 loci whose trees had the lowest robinson-foulds distance (< 0.15; Smith 2020) to the species tree (i.e., genes “4527”, “4848”, “4893”, “4992”, “5264”, “5596”, “5599”, “5620”, “5921”, “6041”, “6320”, and “6924”; combined aligned length 23,413 bases) and concatenated their alignments. We then computed maximum-likelihood branch lengths under a GTR + G model for the ASTRAL topology using RAxML-NG. To also produce ultrametric trees for comparative analyses, we computed branch lengths under a GTR + G model for the ASTRAL topology using BEAST (v.2.6.7., Bouckaert et al. 2019), assuming an uncorrelated lognormal molecular clock (Drummond et al. 2006). To account for branch length uncertainty, we computed 5,000,000 MCMC generations, diagnosed the MCMC using Tracer (Rambaut et al. 2018), removed the outgroup taxa, and thinned the posterior distribution to 100 trees that were used for character reconstructions. To visualize results, we computed a maximum clade credibility tree with median node heights from the posterior, with the root height fixed arbitrarily at 1. We did not attempt to calibrate our tree in absolute time, because the fossil record for Connaraceae is sparse (Streiff 2022) and it was not required for the goals of our study.

### Ancestral character state reconstruction

To infer the evolutionary trajectory of reproductive systems in Connaraceae, we performed two sets of maximum-likelihood analyses using the diversitree package (v.0.9-16, Fitzjohn 2012) of the statistical software R (v.4.2.1, R Core Team 2022). In the first set of analyses, we devised a Markov n-state model (i.e., mkn) of character evolution considering five states: distyly, tristyly, semihomostyly, dioecy and homostyly (Table 1; terminology following Barrett 2019). Species were scored with primary reference to Lemmens (1989a) and Streiff (2022), who relied on careful herbarium observations. Our five-state scoring represents a simplification of Lemmens’ 8-state scoring because we lumped his three types of distyly (Lemmens' types 3, 6, and 7, which differed in whether a middle morph or short morph was lacking, and whether 10 or 5 fertile stamens were present) and his two types of semihomostyly (Lemmens' types 2 and 4, which represents the case when despite sexual organ polymorphism, stigma and anthers are presented at ± the same height). We compared the fit based on AICc (Burnham and Anderson 2002) of three models: (1) a model in which all transition rates were allowed to differ (20 parameters), (2) a symmetrical model in which forward and reverse rates were set as equal (10 parameters), and (3) a model in which all transition rates were equal (1 parameter). Using the parameterization under the best model, we then computed the proportion of likelihood of all states at each internal node (function asr.marginal) under two assumptions: (1) the root states were weighted by the probability of observing the data (i.e., default), and (2) tristyly was set as the root state. We finally tallied the number of transitions between reproductive systems under the best model on the BEAST maximum clade credibility tree, assuming for each internal node the state that resulted in the highest likelihood of the data.

For the second set of analyses, we devised binary scoring (dimorphic and trimorphic; Table 1), to specifically test whether we could reject the hypothesis that trimorphism represented the plesiomorphic state for the family. Here, we scored semihomostyly as trimorphic, as it only occurs in the context of tristyly (Barrett 2019). The reproductive systems homostyly (known only from *Cnestis ferruginea)* and dioecy (known only from *Ellipanthus beccarii*, though functional dioecy may be more widespread, e.g., in *Connarus* spp., Lemmens 1989a) occur too infrequently to be included. We fitted a series of BiSSE models, which have the advantage of being more reliable than mkn models when character states may affect speciation or extinction dynamics (Maddison et al. 2007), as has been shown previously for heterostyly (e.g., de Vos et al. 2014). Although a multistate version of BiSSE is available in the diversitree package, we refrained from using it, because it is too parameter-rich to reliably fit on our modest 35-tip tree. The most complex model we fitted contained six parameters: the transition rates between states, and trimorphic- and dimorphic-specific speciation and extinction rates. We specified a sampling fraction based on an assumed 200 extant Connaraceae species (Streiff 2022). We fitted 5 simplifications of this model by constraining parameters, implementing symmetric transition rates, and/or no extinction, and/or symmetric speciation rates. Each model was fitted to 100 trees from the posterior distribution of the BEAST analysis using maximum likelihood. We compared model fit using AICc (Burnham and Anderson 2002) and reconstructed ancestral states under the best models on the maximum clade credibility tree for visualization. Here, we used the two root constraints as for the mkn-analysis.

## Results

### Phylogeny

The normalized quartet score of the ASTRAL tree was 0.82, indicating that a low to modest amount of incomplete lineage sorting is implied by the pattern of topological conflict and congruence across gene trees. The ASTRAL tree was very well supported (Fig. 2), with all except four nodes receiving a posterior probability of 1.0 (three nodes < 0.99), thus providing a solid basis to evaluate the infrafamilal classification of the family.

Connaraceae is monophyletic in the ASTRAL tree (Fig. 2), and was also monophyletic in 98.5% of the maximum likelihood gene trees (henceforth termed gts, gene tree support); a sister-relation of *Manotes* to Oxalidaceae received minimal support of 1.5% gts. The deepest split in the ingroup was between *Manotes* (gts 98.5%) and the rest of the family (gts 94.9%), with a very long branch between the two Connaraceae clades. The deepest split within the remainder of the family is between a 5-carpelate clade (gts 59.2%) corresponding to Cnestideae in the concept of Lemmens (1989b) and a 1-carpelate clade (gts 70.2%) that contained the paraphyletic Connareae sensu Lemmens (1989b), within which *Jollydora* (i.e., Jollydoreae, gts 95.5%) was nested. Connareae sensu Lemmens (1989b) was not monophyletic in 82.8% of the gene trees either.

The 1-carpellate clade consisting of Connareae sensu Lemmens plus *Jollydora* is well resolved: *Connarus* (of which the four sampled species, representing all continents, formed a monophyletic clade) is sister to *Ellipanthus* plus the representative of *Hemandradenia*; these are jointly sister to *Jollydora* plus its sister group containing *Vismianthus* plus *Burttia*. In contrast, the genera of Cnestideae are in disarray, because *Rourea* in the concept of Breteler (1989) is polyphyletic, and the backbone of this tribe is not resolved. *Rourea* sensu Jongkind (1989) falls in three clades. The first clade comprises *Rourea* species of the former genera *Spiropetalum*, *Paxia*, *Roureopsis, Jaundea* and *Byrsocarpus* that are jointly sister to *Cnestidium*, while the second clade includes *Rourea* species of the former genus *Santaloides*. These two clades plus monophyletic *Cnestis* and *Pseudoconnarus* form a strongly supported clade, but with no backone support (including local posterior probabilies 0.44 and 0.41). In the third clade in which *Rourea* appears, *Rourea parviflora* is sister to *Agelaea*, which are together sister to the rest of Cnestidae. Unfortunately, we were not able to successfully sequence material from the Type species of *Rourea*, *R. frutescens* Aubl. or other American *Rourea* species.

### Evolution of heterostyly

**Fig. 3:**
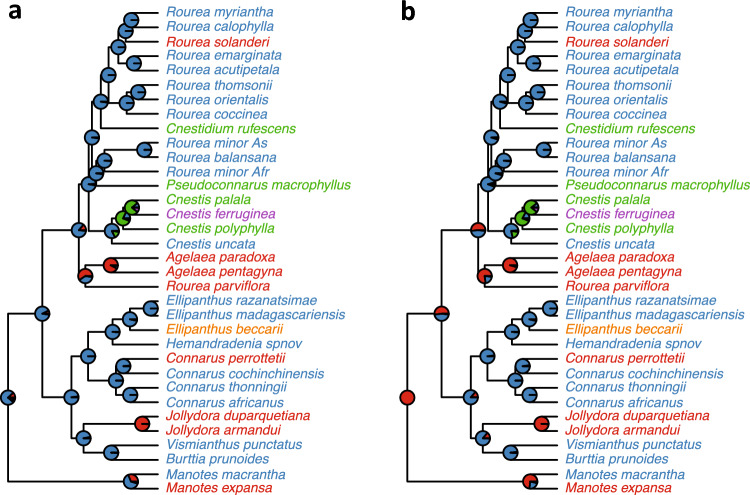
Ancestral character state reconstruction of reproductive systems in Connaraceae under the best-fitting model, employing two root assumptions (**a** default, i.e., root states weighted by their relative probability of observing the data; **b** root fixed at tristyly), using the maximum clade credibility tree from the BEAST analysis. States are indicated by tip colors: blue, distyly; red, tristyly; green, semihomostyly; orange, dioecy; purple, homostyly (see Table 1). Pie charts indicate the proportion of likelihood associated with either ancestral state. Note that the likelihood of either root assumption (**a** and **b**) is equal

The most common reproductive systems in Connaraceae are tristyly and distyly, with single occurrences of dioecy and homostyly, while multiple species are semihomostylous. In the latter reproductive system, style length is polymorphic, but the stigma is placed at the position of one of the two stamen whorls. Tristyly occurs scattered throughout the phylogeny (in both subfamilies and all tribes, see taxonomic treatment) but the species are fewer in number than the distylous species. The 5-state mkn model collapsed to a single transition rate parameter was overwhelmingly strongly supported (AICc 81.70; competing symmetrical model, AICc 97.22; unconstrained model, AICc 169.86). The reconstruction of deeper nodes strongly depends on root assumptions (compare Fig. 3a, b), but in all cases, the number of transitions is rather high (17–18 changes on the maximum clade credibility tree, i.e., on average on every second branch). Because the rate of evolution of tristyly was not approaching zero (but equal to its loss rate in the best model), tristyly may have evolved more than once. Dioecy arose from distyly, and homostyly arose from semihomostyly, in line with our expectations. Overall, our analysis does not reveal a single, most plausible trajectory of reproductive system evolution, rather, the analysis indicates that reproductive system evolution is rather labile in Connaraceae.

To investigate the transitions between di- and trimorphism further, we simplified our scoring to binary and performed a full model selection analysis across a posterior distribution of BEAST trees (Table 2). Here, two models were almost equally supported (Table 2; δAICc 0.15); models differ in allowing for differential speciation rates and agree in having symmetric transition rates, as in the mkn-models above, indicating that we cannot exclude the possibility that tristyly evolved more than once. The speciation rate of trimorphic lineages exceeded its loss rate (λ_0_ > q_01_; Table 2), indicating that tristyly can evolutionarily persist over macroevolutionary timescales even if it evolves rarely. The ancestral state of Connaraceae again remained inconclusive due to a large number of transitions and uncertainty in the rates (Table 2, note wide 95% posterior densities). Overall, even though the deeper nodes remain unclear under all analyses, we find slightly more evidence for a trimorphic root, because the proportion of likelihood associated with that state is higher in 3 out of 4 analyses (Fig. 3). Therefore, a scenario of an origin of tristyly in the ancestor of Connaraceae is congruent with our phylogenetic analysis, but we cannot refute multiple origins of tristyly.

**Table 2:** Model fitting results for ancestral character state reconstructions (state 0: trimorphism; state 1: dimorphism) reporting mean and 95% highest posterior density intervals (in brackets) of ML reconstructions across the posterior distribution of trees from the BEAST analysis

Model constraints	λ_0_	λ_1_	μ_0_	μ_1_	q_01_	q_10_	Implied number of transitions*	Parameternumber	Mean AICc	Mean δAICc
Symmetrical transitions, no extinction (= best model)	3.95 (3.55–4.31)	7.07 (5.71–8.40)	0	0	1.78 (1.34–2.24)		16.1 (13–19)	3	39.65	0
Symmetrical transitions, no extinction, symmetrical speciation	5.96 (5.08–6.87)		0	0	1.46 (1.13–1.74)		15.3 (14–16)	2	39.80	0.15
No extinction	3.23 (2.54–3.96)	7.26 (5.79–8.79)	0	0	1.03 (0.26–1.61)	2.00 (1.31–2.58)	14.6 (12–18)	4	41.62	1.97
No extinction Symmetrical speciation	5.96 (5.08–6.87)		0	0	1.97 (1.36–2.84)	1.50 (0.95–1.79)	15.7 (14–16)	3	42.03	2.38
Symmetrical transitions	3.92 (3.52–4.49)	8.05 (5.71–10.66)	0.04 (0.00–0.00)	1.41 (0.00–3.62)	1.75 (1.33–2.18)		16.0 (13–19)	5	44.93	5.28
No constraints	3.62 (2.42–5.79)	7.42 (5.79–9.30)	1.21 (0.00–5.16)	0.22 (0.00–1.20)	0.74 (0.31–1.52)	2.17 (1.34–2.94)	14.0 (12–18)	6	47.45	7.80

Rates are calibrated in relative time (i.e., root depth equals one)

*Computed by assuming the maximum likelihood state at each internal node

## Discussion

### Revised classification and putative synapomorphies

Although the monophyly of Connaraceae has never been seriously questioned (Breteler 1989), the classification and generic delimitation within the family have been controversial for decades, with widely divergent species and generic concepts across treatments (e.g., Breteler 1989 vs. Forero 1983). Our phylogenetic analysis, which sampled all tribes, all currently accepted genera, and almost all genera synonymized in the last half century, revealed a very well-supported supergeneric backbone, the only unresolved parts pertaining to the backbone of *Rourea* s.l. (Fig. 2). These results thus provide evidence for the efficacy of the Angiosperms353 probe set to obtain high phylogenetic resolution for higher level systematic questions (McDonnell et al. 2021; Baker et al. 2021), though some authors have also demonstrated its power in resolving species radiations and even population level questions (Ottenlips et al. 2021; Thomas et al. 2021; Wenzell et al. 2021). The lack of resolution across the *Rourea* s.l. backbone, on the other hand, is in line with studies failing to resolve higher level clades despite good gene recovery, which may reflect a rapid radiation in the past (e.g., Lee et al. 2021, for Dipsacales). In our case, we recovered a nearly complete species-by-gene matrix (96.2%) indicating that we were successful in recovering ample sequence data from herbarium specimens, underscoring the increasing relevance of herbarium specimens for molecular approaches (Burbano and Gutaker 2023). Moreover, a high standardized quartet score exceeding 0.80 further indicates that the conflict between gene trees and the species tree was not a major issue. Finally, the percentage of gene trees supporting the ASTRAL clades was very high (often exceeding 50%), indicating little of the phylogenetic noise often found in phylogenomic approaches. Our results are therefore amply appropriate for an evaluation of the supergeneric classification of the family, while the resolving the recovered polyphyly of *Rourea* s.l. requires a more targeted, future study with expanded taxon sampling.

Our results are mostly congruent with Lemmens' (1989b) classification in four tribes, but strongly diverge from Schellenberg's (1938) classification (Table 1). Specifically, we recover *Manotes* as clearly distinct (the only genus in Manoteae Lemmens, which contains four or five species; Fig. 1d) and sister to the rest of the family. Its strong support, a comparatively long branch in the ASTRAL tree with ML branch lengths (Fig. 2), and a series of putative vegetative and morphological synapomorphies (see below) allow us to recognize this split newly at the subfamily level (Manotoideae J.M.de Vos & Streiff, subfam. nov.; see taxonomic treatment), as sister to a reinstated Connaroideae that in our circumscription contains all other genera. Connaroideae contains about 200 species and falls into two clades with full support (Fig. 2): the five-carpellate Cnestideae Planch. (ca. 104 spp.) and a uni-carpellate clade that contains the species of Jollydoreae (Gilg) Lemmens and Connareae DC. (ca. 95 spp.). Based on these results, we expand the circumscription of Connareae to include all uni-carpellate Connaraceae (i.e., Connareae plus Jollydoreae sensu Lemmens), and confirm Cnestideae.

There are multiple lines of evidence supporting the decision to create a new subfamily in Connaraceae, Manotoideae, containing *Manotes*. First of all, its phylogenetic position is very distinct, and received full support (Fig. 2). Particularly striking is that the molecular branch lengths separating *Manotes* from Connaroideae greatly exceed the length of the branches between Connaraceae and Oxalidaceae, the closest relative of the family (Fig. 2), and in particular the internal branch lengths in the remainder of the tree. Morphologically, *Manotes* also takes up an isolated position in Connaraceae. Foremost, floral structure differs in that *Manotes* is the only genus in which a well-developed androgynophore occurs. In other Connaraceae, carpels are sessile or at most minutely stipitate (e.g., *Jollydora*), although they may sometimes be briefly united at base (personal observation SJRS on *Rourea* spp.), or even adnate to the androecium (Dickison 1971), but this has not been investigated in detail. Androgynophores occur occasionally throughout eudicots, e.g., in Passifloraceae (Bernhard 1999; de Vos and Breteler 2009), Malvaceae (Brunken and Muellner 2012), Cleomaceae (Bayat et al. 2018), Brunelliaceae and, importantly, also in the closely related Oxalidaceae (Matthews and Endress 2002). Thus, the presence of the androgynophore supports the intermediate phylogenetic position of Manotoideae relative to Oxalidaceae and Connaroideae. The function of the androgynophore may be that by elevating the androecium and gynoecium, a cavity emerges, enclosed by the proximate parts of the petals (which are often postgenitally fused at base, at least in other Connaraceae; Matthews and Endress 2002), from where nectar is less likely to evaporate quickly compared to a higher position in a flower. This could be useful in the obligately outcrossing, heteromorphic flowers of *Manotes* species that rely on appeasing pollinators. For instance, in Grewioideae (Malvaceae) the androgynophore contains nectaries (Brunken and Muellner 2012), and in some Passifloraceae, the cavity created by the androgynophore is surrounded by a nectary ring (sometimes termed annulus, de Vos and Breteler 2009). Indeed, in Connaraceae, nectaries are frequently found at the base of the often basally connate filaments. However, nectary position in *Manotes* was not investigated by Matthews and Endress (2002). Another unusual feature of *Manotes* flowers is their solid styles, without a morphologically evident pollen tube transmission tract (Dickison 1971). Finally, *Manotes* produce fruits with a fleshy arilloid that is elongated in a thread-like structure, from which the seed is pendulous. Although many Connaraceae have seeds hanging partly out of the fruit, such strongly pendulous seeds are otherwise rare in Connaraceae (they occur as well e.g., in *Vismianthus*).

Several vegetative characters also support the distinctness of *Manotes*. Its wood for instance, was considered to have “the most primitive structure” relative to other Connaraceae, because rather than libriform fibers it comprises only or nearly only fiber tracheids and rather abundant parenchyma in long tangential bands, and annual growth rings are absent or at least indistinct (Den Outer and Van Veenendaal 1989). Leaf anatomy is also relatively unusual and allows the identification of *Manotes* at arm's length because it displays a closed venation with the highest order veins in a distinct, very fine, parallel pattern, whereas parallel venation, which also occurs in multiple *Rourea* s.l. species, is typically expressed only at a higher order of venation in the family (Jongkind 1989, personal observation SJRS). Moreover, although few species were invesitgated, seedling architecture appears to deviate from the norm in Connaraceae in that a primary root is absent and many “secondary” (i.e., adventitious) roots develop instead (Breteler 1989). Finally, among the investigated Connaraceae, the predominant cytotype is 2n = 28, whereas only *Manotes* has 2n = 26 (Arends 1989). To conclude, a wealth of characters that span phylo- and cytogenetic and structural characters jointly underpin the distinctness of *Manotes*, and strongly warrant a subfamilial status for this genus.

The remainder of Connaraceae, i.e., Connaroideae, are a clade that is easily distinguished from Manotoideae, as its species have seedlings developing a primary root, mature plants that display growth rings, and with unifoliolate, trifoliolate or pinnate leaves. The flowers do not have a distinct androgynophore. Their follicle fruits have 1 or 2 seeds that are basally to entirely cover by an arilloid. Connaroideae contains two clades that are separated mainly by carpel number: one in Connareae vs. five in Cnestideae. Since five carpels is the norm in Oxalidaceae (Cocucci 2004), Manotoideae and Cnestideae, we consider the single carpel of Connareae to be a derived character. This reflects a trend of carpel reduction, that is in line with the angiosperm-wide trend of reduction in carpel number (Endress 2011). The somewhat deviating pollen and floral morphology of *Jollydora*, previously recognized as its own tribe (Lemmens 1989b) but firmly nested within Connareae (Fig. 2), may thus be considered highly derived within the family, rather than primitive, as Schellenberg (1938) suggested. Our results confirm the circumscription of Cnestideae of Lemmens (1989b), but reveal that the generic recircumscription of Jongkind (1989) did not resolve its polyphyly completely. Although generic recircumscription in this clade requires more dense taxon sampling, the phylogenetic affinity of former genera within *Rourea* is nevertheless morphologically enlightening. For instance, the clades within* Rourea *s.l. containing *Roureopsis*, *Paxia* and *Spiropetalum* on the one hand, and *Byrsocarpus* and *Jaundea* on the other, are jointly characterized by having petals exceeding the length of the calyx two to many more times, the tips of which are frequently folded or rolled inwards, particularly so in the former group. Moreover, these groups of former genera can be distinguished by their different relative arilloid sizes and calyx shapes, among others. This offers good grounds to resolve generic delimitation within Cnestideae in the future.

### Evolution of heterostyly

Connaraceae has long been “dark matter” where the diversity of polymorphic reproductive systems is concerned, with no phylogenetic study addressing its evolution (Barrett 2019), even though its diversity in reproductive systems has long been recognized (Lemmens 1989a). In fact, Lemmens (1989a) discrimintated between eight reproductive systems in the family on morphological grounds, including seven polymorphic ones (named “heterotristyly”; “heterostyly, transitional between heterotristyly and heterodistyly”; “heterodistyly with 10 fertile stamens and short of long styles”; “heterodistyly with rare extreme forms”; “Heterodistyly with 10 fertile stamens and a medium or long styly”; “heterodistyly with 5 fertile stamens”; and “Dioecism”) and homostyly. After careful revision of herbarium material and considering the definitions of Barrett (2019), we could collapse them to five states (Table 1, Fig. 3). This scoring, plus the fact that tristyly occurs scattered throughout the family, offered potential to infer the state from which tristyly may have evolved, which remains poorly understood (Barrett 2019; Charlesworth 1979). Nevertheless, the scoring scheme represents a simplification of quite some variation within the family. For instance, in the *Santaloides* lineage of *Rourea* s.l., the sepals clasp the reproductive organs into a bundle, making the scoring of relative reproductive organ length difficult, whereas later in anthesis or thereafter, the sepals relax and the species appears distylous (Leenhouts 1958; pers. obs. SJRS). Secondly, some clades appear constant in their reproductive system without much variation (e.g. *Agelaea* is always tristylous; the *Byrsocarpus* lineage of *Rourea* s.l. is always distylous), whereas other lineages are highly variably across species (e.g., *Cnestis*, the *Roureopsis* lineage of *Rourea* s.l.). Morover, some of the ancillary characters associated typically with heterostyly may or may not be present, including differences in pollen size and stigmatic surface (da Paz et al. 2024; Lemmens 1989a).

**Fig. 4:**
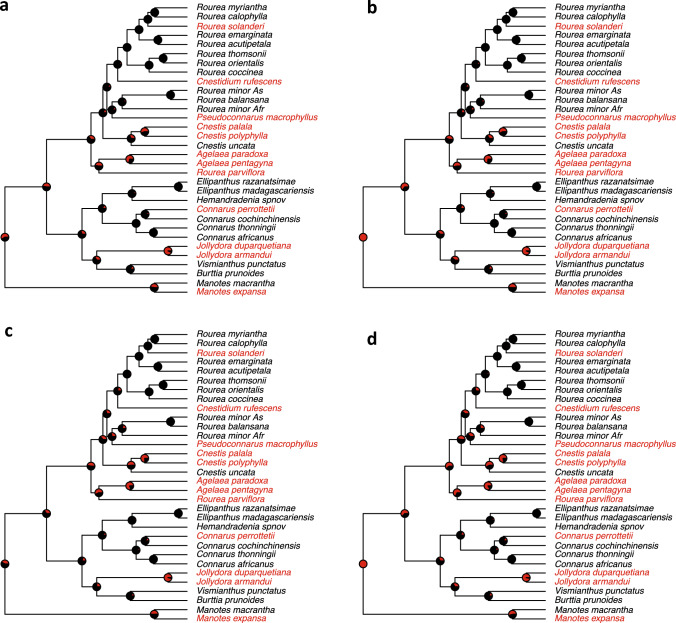
Ancestral character state reconstruction of trimorphism (red) and dimorphism (black) in Connaraceae under the two best-fitting models (**a**, **b** symmetrical transition rates, no extinction; **c**, **d** symmetrical transition and speciation rates, no extinction), and two root assumptions (**a**, **c** default, i.e., root states weighted by their relative probability of observing the data; **b**, **d** root fixed at trimorphic), using the maximum clade credibility tree from the BEAST analysis. Pie charts indicate the proportion of likelihood associated with either ancestral state. Note that binary scoring required semihomostyly to be scored as trimorphic, for it is closely related to tristyly, and homostylous and dioecous species were pruned. Note that under all assumptions, the deeper nodes are equivocal, with a higher likelihood of being trimorphic in 3 out of 4 analyses

Lemmens (1989a) suggested on morphological grounds that tristyly may be ancestral for Connaraceae, while Matthews and Endress (2002) proposed that tristyly may be a synapomorphy for Connaraceae plus Oxalidaceae. Even though our phylogenetic reconstructions of the evolution of heterostyly did not refute a scenario where tristyly is the ancestral state for the whole family, the competing scenario of multiple origins was not conclusively rejected either (Figs. 3, 4; Table 2). Rather than a negative result, these findings illustrate the high lability of reproductive systems in Connaraceae, with ca. 13–19 transitions in reproductive system implied across the phylogenetic tree (Fig. 3; Table 2). This flexibility is underscored by the relatively high transition rate of about one third of the overall speciation rate (Table 2). Phylogenetic studies on the origins of tristyly in other groups are few; in *Narcissus*, tristyly may have evolved once from monomorphism (Graham and Barrett 2004; Pérez et al. 2003); for Pontederiaceae, tristyly evolved once or twice (Kohn et al. 1996), while in Lythraceae, it may have evolved up to 5 times (Morris 2007). For Connaraceae, we propose that the most likely scenario is one of multiple origins of tristyly, involving an initial origin that was retained in *Manotes*, *Agelaea*, and possibly *Jollydora*, while other cases of tristyly may represent re-gains of tristyly after initial losses (e.g., for *Connarus perrottetii,* and *Rourea solanderi*; Fig. 3). Unfortunately, our ancestral character state reconstructions remain inconclusive regarding the exact number of gains of tristyly (Figs. 3, 4). However, that we have multiple origins of such a rare reproductive system within the same family is reminiscent of the situation for distyly, that in several lineages evolved multiple times in paralell (e.g., Primulaceae, de Vos et al. 2014; Boraginaceae; Cohen 2014; *Nymphoides*, Tippery and Les 2011).

We do find clear evidence that distyly arose multiple times from tristyly (Figs. 3, 4), either through loss of the mid morph (as reported for other systems, Barrett 2019) or loss of the short-style morph (e.g., in *Connarus*, Lemmens 1989a). Transitions of tristyly to distyly are more commonly found, e.g., in *Oxalis* (Gardner et al. 2012), *Pemphis* (Lewis and Rao 1971) and *Lythrum* section *Euhyssopifolia* (Ornduff 1979). Tristyly is generally thought to be genetically controlled by two loci, termed S and M (Barrett 2019; Charlesworth 1979), rather than a single S-locus as for distyly (e.g., Potente et al. 2022). Given these patterns, it is therefore unsurprising that the breakdown of tristyly at a molecular level may involve multiple, independent sets of mating-system modifier genes (Arunkumar et al. 2017), while at a population level, the loss of tristyly may be initiated by demographic deviations from isoplethy (i.e. equal morph ratios; Barrett 2019).

Underscoring the richness of reproductive systems in Connaraceae, we find a phylogenetic sequence from tristyly to distyly to semihomostyly to homostyly in the ancestors of *Cnestis ferruginea* (which has characteristics typically of a selfer, such as small flowers, no herkogamy, and large geographic distribution) and a sequence from tristyly to distyly to dioecy in the ancestors of *Ellipanthus beccari* (Fig. 3), generally in line with other studies (Barrett 2019). Strikingly, even our 5-character coding represents a simplification of the real situation (Lemmens 1989a). For instance, *Rourea* s.l. is stated to be always distylous (Jongkind 1989), while a reevaluation of herbarium specimens suggest that at least *Rourea solanderi* is tristylous (personal observation SJRS). However, many species remain under-collected, poorly identified due to outdated and scattered taxonomic treatments, and contain small flowers, making it often challenging to determine what flower morph is present in what species. Moreover, much basic data remains lacking, such as on physiological incompatibility systems and ancillary characters. According to Baker (1962) *Rourea coccinea* is largely, but not completely, self-incompatible; Lemmens (1989a) found no evidence for morph-specific pollen grain size. Nevertheless, given the sister relation of Connaraceae and Oxalidaceae, where tristyly also occurs, and is frequently lost, the two families together represent a hot spot of stylar polymorphism evolution with great potential for studying the evolution of reproductive systems.

## Conclusions

Connaraceae is arguably the plant family with the most diverse array of reproductive polymorphisms, yet taxonomic and systematic confusions rendered it inaccessible for studies at macroevolutionary scales. Our approach of sampling representative species for nomenclatural lineages (rather than sampling representatives of genera based on a biological concept), all from herbarium specimens, yielded a very well-supported phylogenetic tree, which enabled us to confidently revise the supergeneric classification of the family. The only poorly resolved part of the tree involves the polyphyly of *Rourea* s.l. It is striking that this pantropical genus, with the most confusing taxonomic history, also turns out to be the most phylogenetically challenging group within the family. Overall, our study underlines that technical progress in “herbariomics” enables the use of herbarium specimens much beyond the scope for which they were originally collected, effectively resolving challenging systematic problems (Burbano and Gutaker 2023; De Vos and Stöcklin 2024). In the specific case of our reconstructions of the trajectory of evolution of heterostyly, this approach has revealed a striking lability of the various reproductive polymorphisms, which is particularly valuable and timely given recent progress in understanding the molecular genetics of heterostyly and availability of whole genomes of heterostylous species (Barrett 2019; Potente et al. 2022). Our study represents a first, necessary step to provide a robust systematic framework to unlock Connaraceae for further such studies.

### Taxonomic treatment

**Manotoideae** J.M.de Vos & Streiff, **subfam. nov.** ≡ *Manoteae* Lemmens, Agricultural University Wageningen Papers 89(6): 116. 1989.

*Diagnosis*: Lianas, with seedlings lacking development of a primary root, many accessory roots developing instead, mature plants with leaves pinnate, with characteristic parallel scalariform terminal veinlets, growth rings absent in wood but metatracheal parenchyma bands present, flowers with 5 carpels borne on a distinct androgynophore, follicle restricted at base, 1-seeded, seed enveloped in an arilloid, attaching to the base of the follicle.

*Type genus*: *Manotes* Sol. ex Planch.

*Genera included*: *Manotes* Sol. ex Planch. (4–5 spp.)

*Distribution*: West- and Central tropical Africa.

*Note*: Manotoideae is a newly recognized subfamily, because it differs profoundly from Connaroideae in a range of vegetative and reproductive characters (see main text for discussion) and it is phylogenetically isolated.

**Connaroideae** Gilg, Nat. Pflanzenfam. Nachtr. 1: 189. 1897.

*Type genus*: Connarus L. Sp. Pl. 2: 675. 1753.

*Included tribes*: Connareae DC., Cnestideae Planch.

*Diagnosis*: Lianas, shrubs or small trees, with seedlings developing a primary root, mature plants with unifoliolate, trifoliolate or pinnate leaves, growth rings usually present, flowers with 1 or 5 carpels, androgynophore not distinct, follicle with 1 or 2 seeds, basally to entirely covered by an arilloid.

**Tribes within Connaroideae**

**Connareae** DC.

 = Jollydoreae Lemmens Agricultural. University. Wageningen Papers 89(6): 116. 1989, **syn. nov.**

*Type genus*: *Connarus* L.

*Genera included*: *Burttia* Baker f. & Exell (1 sp.), *Connarus* L. (c. 80 spp.), *Ellipanthus* Hook.f. (6 spp.), *Jollydora* Pierre ex Gilg (3 spp.), *Hemandradenia* Stapf (ca. 3 spp.), *Vismianthus* Mildbr. (2 spp.)

*Distribution*: pantropical.

*Note*: Connareae contains all 1-carpellate Connaraceae. Morphologically, *Jollydora* remains somewhat distinct (e.g., 2-seeded rather than 1-seeded fruits, tetracolpate pollen rather than typically tricolporate pollen) leading previous authors to recognize it at tribal (Lemmens 1989b) or even subfamilial (Schellenberg 1938) level. However, the consequence of recognizing it supergenerically, given its nested phylogenetic position, would be that *Vismianthus* plus *Burttia* require the same status, which is not warranted by their close morphological relation to the other genera of Connareae. Therefore, we consider *Jollydora* to be a genus with more derived character states within a broader circumscribed Connareae. This has the convenient consequence that all 1-carpellate Connaroideae (and thus all 1-carpellate Connaraceae) belong to a single tribe, Connareae.

**Cnestideae** Planch.

*Type genus*: *Cnestis* Juss.

*Genera included*: *Agelaea* Sol. ex Planch. (8 spp.), *Cnestis* Juss. (13 spp.), *Pseudoconnarus* Radlk. (5 spp.), *Rourea* Aubl. s.l. (ca. 78 spp.; but polyphyletic, probably to be split into *Byrsocarpus* Schumach. (4 spp.), *Rourea* Aubl. [incl. *Bernardinia* Planch. and *Cnestidium* Planch.]. (ca. 50 spp.), *Roureopsis* Planch. [incl. *Spiropetalum* Gilg and *Paxia* Gilg] (14 spp.), *Santaloides* G.Schellenb. (ca. 9 spp.), and *Santaloidella* G.Schellenb. (1 sp.).

*Distribution*: pantropical.

*Note*: Cnestideae contains all 5-carpellate Connaroideae. The list of included genera is tentative and the subject of ongoing work. Nevertheless, although the phylogenetic backbone of Cnestideae is poorly supported, the polyphyly of *Rourea* s.l. is evident. The present concept of *Rourea* s.l. is therefore untenable, but revising generic delimitation requires denser taxon sampling.

### Information on Electronic Supplementary Material

**Online Resource 1.** Genbank accession numbers of sequences used during the HypPiper pipeline for non-Angiosperms353 loci.

**Online Resource 2.** Alignments and RAxML gene trees for all included loci; ASTRAL species tree based on all RAxML gene trees; ASTRAL species tree with branch lengths based on a concatenated set of loci.

### Supplementary Information

Below is the link to the electronic supplementary material.

Supplementary file1 (DOCX 16 kb)

Supplementary file2 (ZIP 1968 kb)

## Data Availability

Raw genetic data (reads) are publicly available under persistent identifiers as stated in Table 1. Analyzed genetic data (alignments per locus) and phylogenetic data (gene and species trees) are available as Online Supplementary Data S1.
