# Supplementary material for: Phylogenomics of the pantropical Connaraceae: revised infrafamilial classification and the evolution of heterostyly
Source: Plant Syst Evol. 2024 Aug 3;310(4):29. doi: 10.1007/s00606-024-01909-y (PMC11297820; doi:10.1007/s00606-024-01909-y)
Supplement: Supplementary file 1 — Supplementary file1 (DOCX 16 kb) [file 606_2024_1909_MOESM1_ESM.docx]

Table S1. Genbank accession numbers for non-Angiosperm353 sequences used as "bait" sequences during locus assembly using Hybpiper.

| **Locus** | **Species** | **GenBank Accession No.** |
| --- | --- | --- |
| Nuclear: Ribosomal DNA (ITS) | *Biophytum sp.* | EU436865.1 |
|  | *Rourea minor* | JQ230983.1 |
| Nuclear: Ribosomal DNA | *Averrhoa carambola* | KU569491.1 |
| Chloroplast: matK | *Agelaea macrophylla* | MG968352.1 |
|  | *Biophytum sensitivum* | OL689899.1 |
|  | *Cnestidium rufescens* | KJ593828.1 |
|  | *Connarus semidecandrus* | MH332535.1 |
|  | *Ellipanthus tomentosus* | KJ708923.1 |
|  | *Oxalis acetosella* | LC617149.1 |
|  | *Rourea asplenifolia* | MG968348.1 |
|  | *Rourea glabra* | JQ587280.1 |
|  | *Rourea surinamensis* | KJ012756.1 |
| Chloroplast: RbcL | *Agelaea borneensis* | MG968277.1 |
|  | *Agelaea macrophylla* | MG968270.1 |
|  | *Averrhoa carambola* | OL536763.1 |
|  | *Biophytum sensitivum* | OL536778.1 |
|  | *Cnestidium rufescens* | KJ594183.1 |
|  | *Connarus conchocarpus* | L29493.2 |
|  | *Connarus semidecandrus* | MH332412.1 |
|  | *Ellipanthus tomentosus* | KJ594701.1 |
|  | *Jollydora duparquetiana* | KC628036.1 |
|  | *Oxalis acetosella* | LC617116.1 |
|  | *Oxalis latifolia* | EU002282.1 |
|  | *Rourea asplenifolia* | MG968263.1 |
|  | *Rourea glabra* | JQ594752.1 |
|  | *Rourea minor* | FJ707537.1 |
|  | *Rourea surinamensis* | KJ082548.1 |
| Chloroplast: trnK-matK | *Oxalis dentata* | EU437341.1 |
|  | *Oxalis tenella* | EU437354.1 |
|  | *Oxalis tomentosa* | EU437358.1 |
